# Imlunestrant in ER+ advanced breast cancer – bridging innovation and clinical reality

**DOI:** 10.1097/MS9.0000000000004417

**Published:** 2025-12-03

**Authors:** Aleeza Khalid, Momina Inayat, Muddassir Khalid

**Affiliations:** Department of Medicine, Nishtar Medical University, Multan, Pakistan

## Introduction: breast cancer, heterogeneity, and the challenge of resistance

Globally, breast cancer continues to be a major cause of cancer-related morbidity and death. It is responsible for about 15% of female cancer deaths and nearly one-third of all cancers in women. Using immunohistochemistry, the biological and clinical behavior is further divided into subtypes according to the expression of the human epidermal growth factor receptor-2 (HER2), progesterone receptor (PR), and estrogen receptor (ER). Approximately 70% of tumors are HER2-negative and hormone receptor-positive (HR+/HER2–), 15–20% are HER2+, and 15% are triple-negative (HR–/HER2–)^[1]^.

Estrogen signaling plays a key role in the growth and survival of tumors in HR+ disease. By binding to the nuclear ER (encoded by ESR1), estrogen controls transcription by interacting with growth factor pathways and estrogen response elements. Exogenous estrogen exposure, endocrine instability, or dysregulation of the estrogen/progesterone balance can all promote uncontrolled proliferation, the buildup of DNA damage, and stromal remodeling, all of which favor metastasis and progression^[2,3]^.

Endocrine therapy (ET) has been the backbone of ER+/HER2– breast cancer treatment, with agents such as tamoxifen (a selective estrogen receptor modulator) and aromatase inhibitors (AIs) forming mainstays in both early and advanced settings. Fulvestrant, a selective estrogen receptor degrader (SERD), was the first approved agent that not only antagonizes ER but promotes its proteasomal degradation. However, many patients eventually develop resistance, usually mediated by on-target ESR1 mutations^[4]^.

## ESR1 gene mutations, fusions, and endocrine resistance

When treating advanced HR+ breast cancer, resistance to ET is an unavoidable hazard. The emergence of ESR1 ligand-binding domain (LBD) point mutations, most commonly at codons 537 and 538, is a major mechanism of acquired resistance. These mutations confer ligand-independent constitutive activation of ER, attenuate response to tamoxifen or AIs, and reduce sensitivity to wild-type-targeted SERDs (e.g., fulvestrant, giredestrant). Under the selective influence of previous ET, these mutations are more prevalent in metastatic samples^[5]^.

Another emerging mechanism is ESR1 fusion proteins. Though very rare, ESR1 fusions may mark particularly aggressive, resistant phenotypes and represent an underexplored therapeutic target^[6]^.

Because of the importance of ER signaling and the ability of cancer cells to adapt under endocrine pressure, the field has looked for next-generation SERDs, and several other modalities to overcome LBD mutations and bypass resistance^[7]^.Among these, imlunestrant (LY3484356) has drawn interest lately as a potentially effective oral SERD with brain-penetrant qualities and strength against mutant ER.

## Imlunestrant: pharmacology, preclinical rationale, and early development

Imlunestrant is an oral, selective estrogen receptor antagonist/degrader designed to break down the ER and stop its transcription, even when ESR1 is mutated. Prior research showed that imlunestrant outperformed fulvestrant in head-to-head models by efficiently degrading both wild-type and mutant ER (e.g., Y537S), inhibiting growth in ER+ cell lines with ESR1 mutations, and inducing tumor regression in patient-derived xenograft models with Y537S mutations^[8]^.

The early clinical development (Phase Ia/Ib, EMBER study) established the recommended phase II dose, characterized pharmacokinetics (PKs), safety, and preliminary activity. Imlunestrant showed good PK characteristics, prolonged exposures above the maximum concentrations of fulvestrant, and tolerable toxicity that was mostly restricted to minor side effects like gastrointestinal symptoms^[9]^.

The large Phase III trial, EMBER-3 (NCT04975308), was made possible by these positive results. Its purpose was to compare standard-of-care (SOC) ET (fulvestrant or exemestane) with imlunestrant (monotherapy) or imlunestrant plus abemaciclib in patients with ER+, HER2-advanced breast cancer who had previously received AIs, either with or without prior CDK4/6 inhibitors^[10]^.

## EMBER-3: efficacy, safety, and patient-reported outcomes

In the phase III EMBER-3 trial, patients with ER-positive, HER2-negative advanced breast cancer were randomly assigned 1:1:1 to receive imlunestrant (400 mg daily), SOC ET, or combination arm (imlunestrant plus abemaciclib)^[11]^.

Immunoestrant monotherapy reduced progression or death in ESR1-mutant patients by 38%, improving median progression-free survival (PFS) to 5.5 months compared to 3.8 months with SOC. The PFS was 5.6 vs. 5.5 months in the entire population (including the ESR1 wild-type), indicating a numerical but non-significant improvement.

Imlunestrant plus abemaciclib produced a PFS of 9.4 months as opposed to 5.5 months with imlunestrant alone indicating a synergistic benefit regardless of ESR1 status.

This shows that it is particularly effective in patients with ESR1 mutations.

However, imlunestrant and standard therapy had similar overall treatment-emergent adverse events (83% vs. 84%), with fewer grade ≥3 events (17% vs. 21%). Treatment-emergent adverse events of grade ≥3 rose to 49% in combination regimens. Whereas combination therapy demonstrated higher rates of diarrhea (86%), nausea (49%), and neutropenia (48%), all of which were primarily low grade, fatigue (23%), diarrhea (21%), and nausea (17%) were the most common any-grade events with imlunestrant. There were very few discontinuations (4% with monotherapy, ~6% with combination), and dose reductions were uncommon (2%) with the exception of combination (39%). Bradycardia, interstitial lung disease, thromboembolism, and elevated liver enzymes were among the rare serious toxicities.

According to patient-reported outcomes from EMBER-3, imlunestrant (either alone or in combination with abemaciclib) preserved or slightly enhanced physical function and global health status/quality of life, particularly in the ESR1-mutant subgroup, in contrast to the declines observed with SOC ET^[12]^.

Yet, 20–40% of patients at higher clinical risk experience distant recurrence within 5 years despite optimal ET. Since imlunestrant has shown PK exposure greater than that of fulvestrant and promising early efficacy signals, the current phase 3 EMBER-4 trial is comparing imlunestrant to the adjuvant ET prescribed by the physician (tamoxifen or AI) for about 6000 patients with ER+, HER2-EBC who have finished 2–5 years of standard adjuvant ET but are still at higher risk of recurrence. Key secondary endpoints include overall survival, PKs, safety, patient-reported outcomes, distant relapse-free survival, and invasive disease-free survival, which is the main endpoint^[13]^.

## Advantages and unmet needs addressed by imlunestrant

Imlunestrant fills in a number of long-standing endocrine treatment gaps. More patient convenience and adherence are provided by its oral bioavailability, which gets around the discomfort and logistical problems of intramuscular fulvestrant. The notable benefit of PFS in ESR1-mutant disease indicates that it can get past a key resistance mechanism that restricts the use of existing treatments. Furthermore, its better patient-reported results and favorable safety profile demonstrate tolerability appropriate for long-term use. Because the central nervous system (CNS) is a sanctuary site that is frequently resistant to conventional endocrine agents, preclinical evidence of blood–brain barrier penetration increases the likelihood of controlling or preventing CNS metastases. Its potential as a backbone for combination strategies is further supported by the synergistic efficacy seen with abemaciclib in EMBER-3, regardless of ESR1 status. Lastly, the Guardant360 CDx assay’s concurrent approval guarantees accurate patient selection for ESR1-mutant disease, which is a major step toward customized ET. All of these benefits put imlunestrant in a position to be a next-generation SERD that treats advanced HR-positive breast cancer by bridging the gap between effectiveness, convenience, and precision.

## Potential challenges, unanswered questions, and future directions

Despite its potential, imlunestrant’s enthusiasm is tempered by a number of issues. Its main benefit might be limited to ESR1-mutant disease rather than acting as a universal substitute for current ETs, as the improvement in PFS in the entire EMBER-3 population was numerically favorable but not statistically significant. The combination with abemaciclib was linked to significantly higher rates of grade 3 adverse events, raising concerns about tolerability in routine practice, particularly for older or comorbid patients, even though toxicity with monotherapy is still manageable. Clinical efficacy against CNS metastases is still unknown, despite preclinical research suggesting blood–brain barrier penetration. Furthermore, the need for improved diagnostic techniques is highlighted by the possibility that heterogeneity among ESR1 mutations and fusions could affect drug sensitivity and make biomarker-driven patient selection more difficult. Lastly, concerns about cost, accessibility, and diagnostic availability may limit imlunestrant’s global adoption, especially in low- and middle-income areas. To determine its role across treatment lines and verify whether early efficacy signals result in significant survival and quality-of-life gains, long-term data from ongoing studies, including EMBER-4 and beyond, will be crucial.

## Conclusion

Imlunestrant, a strong and oral substitute for fulvestrant that may be able to overcome ESR1-mediated resistance, is a significant development in the rapidly developing field of ET. While ongoing research like EMBER-4 aims to define its preventive role in early-stage disease, early clinical trials such as EMBER-3 highlight its effectiveness, especially among ESR1-mutant tumors. However, there are still remaining enquiries regarding cost-effectiveness, long-term results, and the best sequencing with other targeted agents. The development of clinical data suggests that immunoestrant may not only improve the treatment of endocrine-resistant breast cancer but also change our understanding of OR signaling in general, indicating a step closer to genuinely customized ET.

TITAN Guidelines: This manuscript is in compliant to the TITAN Guidelines, 2025, declaring no use of artificial intelligence^[14]^.

## Data Availability

Data available on request from the authors.
